# Secondary central nervous system involvement in patients with diffuse large B-cell lymphoma treated with rituximab combined CHOP therapy – a supplementary analysis of JCOG0601

**DOI:** 10.1007/s00277-024-05620-3

**Published:** 2024-01-27

**Authors:** Kazuyuki Shimada, Ken Ohmachi, Ryunosuke Machida, Shuichi Ota, Hidekazu Itamura, Hideki Tsujimura, Nobuyuki Takayama, Takaki Shimada, Mitsutoshi Kurosawa, Takayuki Tabayashi, Tatsu Shimoyama, Koichi Ohshima, Kana Miyazaki, Dai Maruyama, Tomohiro Kinoshita, Kiyoshi Ando, Tomomitsu Hotta, Kunihiro Tsukasaki, Hirokazu Nagai

**Affiliations:** 1 Department of Hematology and Oncology, Nagoya University Graduate School of Medicine, 65 Tsurumai-Cho, Showa-Ku, Nagoya, Aichi 466-8550 Japan; 2 Department of Hematology and Oncology, Tokai University School of Medicine, Kanagawa, Japan; 3 Japan Clinical Oncology Group Data Center, National Cancer Center Hospital, Tokyo, Japan; 4 Department of Hematology, Sapporo Hokuyu Hospital, Sapporo, Japan; 5 Division of Hematology, Respiratory Medicine and Oncology, Department of Internal Medicine, Faculty of Medicine, Saga University, Saga, Japan; 6 Department of Hematology-Oncology, Chiba Cancer Center, Chiba, Japan; 7 Department of Hematology, Faculty of Medicine, Kyorin University, Mitaka, Japan; 8 Division of Clinical Oncology/Hematology, Department of Internal Medicine, The Jikei University School of Medicine, Tokyo, Japan; 9 Department of Hematology, National Hospital Organization Hokkaido Cancer Center, Sapporo, Japan; 10 Department of Hematology, Saitama Medical Center, Saitama Medical University, Kawagoe, Japan; 11 Department of Medical Oncology, Tokyo Metropolitan Cancer and Infectious Diseases Center Komagome Hospital, Tokyo, Japan; 12 Department of Pathology, Kurume University School of Medicine, Kurume, Japan; 13 Department of Hematology and Oncology, Mie University Graduate School of Medicine, Tsu, Japan; 14 Department of Hematology Oncology, Cancer Institute Hospital, Japanese Foundation for Cancer Research, Tokyo, Japan; 15 Department of Hematology and Cell Therapy, Aichi Cancer Center Hospital, Nagoya, Japan; 16 Department of Hematology, National Hospital Organization Nagoya Medical Center, Nagoya, Japan; 17 Department of Hematology, National Cancer Center Hospital East, Kashiwa, Japan

**Keywords:** Central nervous system-international prognostic index, Diffuse large B-cell lymphoma, International prognostic index, JCOG0601, Secondary central nervous system involvement

## Abstract

**Abstract:**

Secondary central nervous system involvement (sCNSi) in diffuse large B-cell lymphoma (DLBCL) is fatal. However, its features in patients with sCNSi who are categorized as lower risk by international prognostic index (IPI) or CNS-IPI are not yet fully understood. In the present analysis, we evaluated DLBCL patients who developed sCNSi at their first progression and who participated in JCOG0601, most of whom were lower risk by IPI. Of 409 patients, 21 (5.1%) developed sCNSi during a median follow-up of 4.9 years. Five-year cumulative incidence of sCNSi were 5.1%; and 4.0%, 5.3%, and 11.5% at low, intermediate, and high risk of CNS-IPI, respectively. The most common locations of extranodal lesions at the time of registration in patients with sCNSi were the stomach (n = 4), paranasal cavity (n = 3), and bone marrow (n = 2). In univariable analysis, paranasal cavity lesion was a high-risk factor for sCNSi (subdistribution hazard ratio, 4.34 [95% confidence interval 1.28–14.73]). Median overall survival after sCNSi was 1.3 years, with a 2-year overall survival rate of 39.3%. The incidence of sCNSi in DLBCL patients at lower risk of CNS-IPI was low, as previously reported, but paranasal cavity lesion might indicate high risk for organ involvement.

**Clinical trial registration:**

JCOG0601 was registered in the UMIN Clinical Trials Registry (UMIN000000929, date of registration; December 04, 2007) and the Japan Registry of Clinical Trials (jRCTs031180139, date of registration; February 20, 2019).

**Supplementary Information:**

The online version contains supplementary material available at 10.1007/s00277-024-05620-3.

## Introduction

Secondary central nervous system involvement (sCNSi) develops in approximately 5% of patients with diffuse large B-cell lymphoma (DLBCL) [1–5]. However, the risk is as high as 40% in certain high-risk patients, such as those harboring *MYC/BCL2* dual translocation with or without *BCL6* translocation or MYC/BCL2 dual expression, and in those with kidney and/or adrenal gland involvement [6–8]. CNS prophylaxis is applied to these high-risk patients in clinical practice; however, the role of CNS prophylaxis in DLBCL is currently under debate [9–13].

Despite the limited overall risk of sCNSi in DLBCL patients, attention is paid to sCNSi for the reason that the prognosis is extremely poor, with a median overall survival (OS) after the development of sCNSi of only a few months [9]. Among the many efforts to avoid this complication, a representative solution to prevent sCNSi in a specific subtype of DLBCL is that for primary testicular lymphoma. Regarding the high risk of sCNSi even in limited-stage primary testicular lymphoma patients, as reported in a retrospective analysis, the application of intrathecal chemotherapy and contralateral testicular irradiation displayed a dramatic reduction of the risk in a phase 2 trial [14, 15]. For intravascular large B-cell lymphoma, a peculiar subtype of DLBCL with high risk of sCNSi, standard immunochemotherapy combined with CNS-directed therapy including high-dose methotrexate and intrathecal chemotherapy also revealed better outcomes in a recent phase 2 trial than in historical controls [16, 17]. The success shown in these specific subtypes implies that an appropriate approach would be useful for reducing the risk of sCNSi; however, among patients considered to be lower risk according to currently used predictive indexes, the characteristics of those who developed sCNSi are largely unknown.

JCOG0601 is a randomized phase 2/3 trial that compared standard R-CHOP-21 with RW-CHOP-21, in which rituximab was administered weekly 8 times from the commencement of treatment in untreated DLBCL patients without CNS involvement [18]. Most of the patients participating in JCOG0601 were categorized as lower risk by the international prognostic index (IPI), which means that most patients were also in the lower risk category of CNS-IPI [19, 20]. The primary analysis revealed that RW-CHOP was not superior to standard R-CHOP. As no CNS-directed therapy was allowed in JCOG0601, the JCOG0601 cohort is therefore suitable for investigation of subsequent CNS events. The present analysis was thus conducted to elucidate the characteristics of sCNSi in patients mainly at lower risk of sCNSi according to current predictive indices.

## Patients and Methods

### JCOG0601

JCOG0601 (jRCTs031180139) was a phase 2/3 study conducted by the Lymphoma Study Group of Japan Clinical Oncology Group (JCOG-LSG). The study compared standard R-CHOP-21 (arm A) with RW-CHOP-21 (arm B), in which rituximab (375 mg/m^2^) was administered weekly 8 times from the commencement of treatment. The primary endpoint of the phase 2 part was the investigator-assessed complete response (CR) rate in arm B, and that of the phase 3 part was progression-free survival (PFS) with secondary endpoints including OS and adverse events. At the beginning of the study, only DLBCL patients without CNS involvement with advanced stage and a lower IPI risk were eligible, and the protocol was amended 33 months later to permit the enrollment of patients with any IPI risk and any clinical stage because of poor accrual. No CNS-directed therapy was allowed in the study treatment. Of the 423 patients enrolled in the JCOG0601, 409 patients were eligible, and these were analyzed in the present study.

### Outcomes and statistical methods

Patients who developed CNS involvement at their first progression or relapse were considered to have sCNSi. Data regarding the site of disease at the time of registration to JCOG0601 and at the time of progression or relapse were collected from case report forms. The site of disease was determined by investigators at each institution, and a central review of the radiological/cytological findings was not performed. Information regarding parenchymal or meningeal relapse was not strictly collected. Central monitoring was performed to ensure that the study was being carried out properly. Histological diagnosis before enrollment in the study was centrally reviewed by the study-specific pathologist panel. OS after progression was defined as the date from the first progression or relapse to any cause of death.

The cumulative incidence function of sCNSi was estimated with consideration of competing risks of death and non-CNS progression or relapse. The cumulative incidence function was compared among CNS-IPI groups (high vs. intermediate vs. low) and between treatment arms (arm A vs. arm B) by Gray’s test. OS after progression were estimated by the Kaplan–Meier method and compared between the sCNSi group and the non-CNSi progression or relapse group by log-rank test. In univariable analysis, subdistribution hazard ratio (sHR) was estimated by the Fine–Gray mode to assess the effect on the incidence of sCNSi of factors including age, sex, performance status, lactate dehydrogenase, clinical stage, extranodal sites, the presence of “B” symptoms, cell of origin determined by Hans’ criteria, and the sites of disease at registration (orbital cavity, paranasal cavity, bone marrow, breast, bone, kidney, adrenal gland, and testis). All statistical analyses were performed using SAS ver. 9.4.

## Results

### Patients

Table 1 lists the patient characteristics. In the 409 patients, the median age of both arms was 62 years, and 222 (54%) patients were older than 60 years. Two hundred twenty-seven (56%) patients were male and 335 (82%) patients were categorized as lower IPI risk. According to CNS-IPI, there were 203 (50%), 180 (44%), and 26 (6%) patients in the low, intermediate, and high risk groups, respectively. More than one extranodal lesion was observed in 43 (11%) patients, and 31 (8%) patients had bone marrow involvement. In terms of organs indicated as high risk for sCNSi by CNS-IPI, 5 (1%) patients and 9 (2%) patients had disease in kidney and adrenal gland, respectively.

**Table 1:** Patient characteristics

		Total (n = 409)		Arm A (n = 204)		Arm B (n = 205)	
		n	%	n	%	n	%
Age (y)	Median	62	-	61	-	62	-
	> 60	222	54	111	54	111	54
Sex	Male	227	56	112	55	115	56
PS	> 1	12	3	6	3	6	3
LDH	> ULN	192	47	108	53	84	41
Stage	III or IV	188	46	105	51	83	40
Ext N	> 1	43	11	28	14	15	7
	Orbital cavity	2	0	0	0	2	1
	Paranasal cavity	16	4	9	4	7	3
	Bone marrow	31	8	17	8	14	7
	Breast	8	2	5	2	3	1
	Kidney	5	1	4	2	1	0
	Adrenal gland	9	2	5	2	4	2
	Bone	24	6	12	6	12	6
B symptoms present	51	12	24	12	27	13	
COO	GCB	123 of 333	37	62 of 171	36	61 of 162	38
IPI	Low	204	50	92	45	112	55
	Low-intermediate	131	32	64	31	67	33
	High-intermediate	51	12	33	16	18	9
	High	23	6	15	7	8	4
CNS-IPI	Low	203	50	91	45	112	55
	Intermediate	180	44	96	47	84	41
	High	26	6	17	8	9	4

Abbreviations: PS, performance status; LDH, lactate dehydrogenase; Ext N, extranodal; ULN, upper limit of normal; COO, cell of origin; GCB, germinal center B-cell; CNS, central nervous system; IPI, international prognostic index

### Secondary CNS involvement

**Fig. 1:**
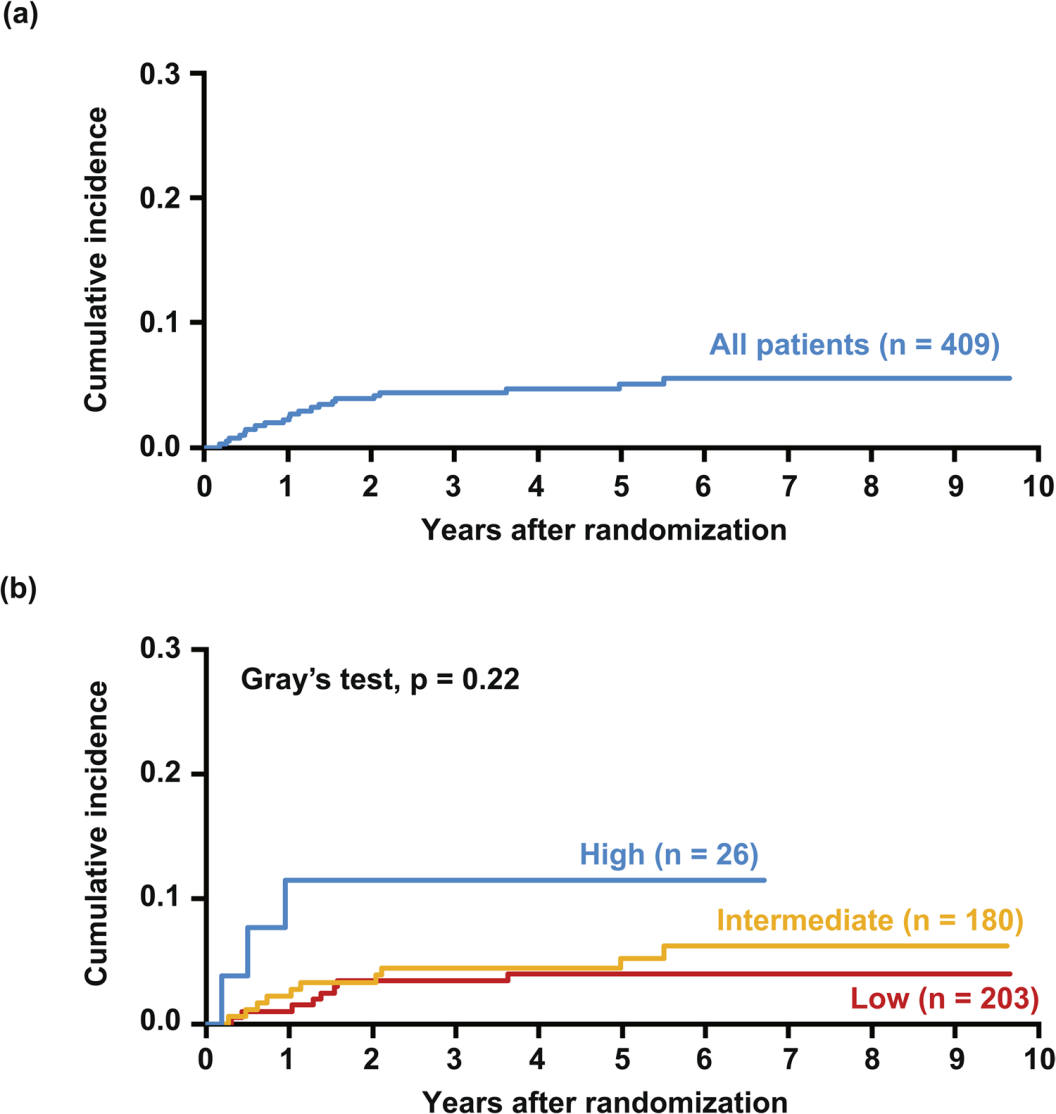
Cumulative incidence of sCNSi in all patients. The cumulative incidence of sCNSi is shown in all patients (**a**) and according to CNS-IPI (**b**)

**Fig. 2:**
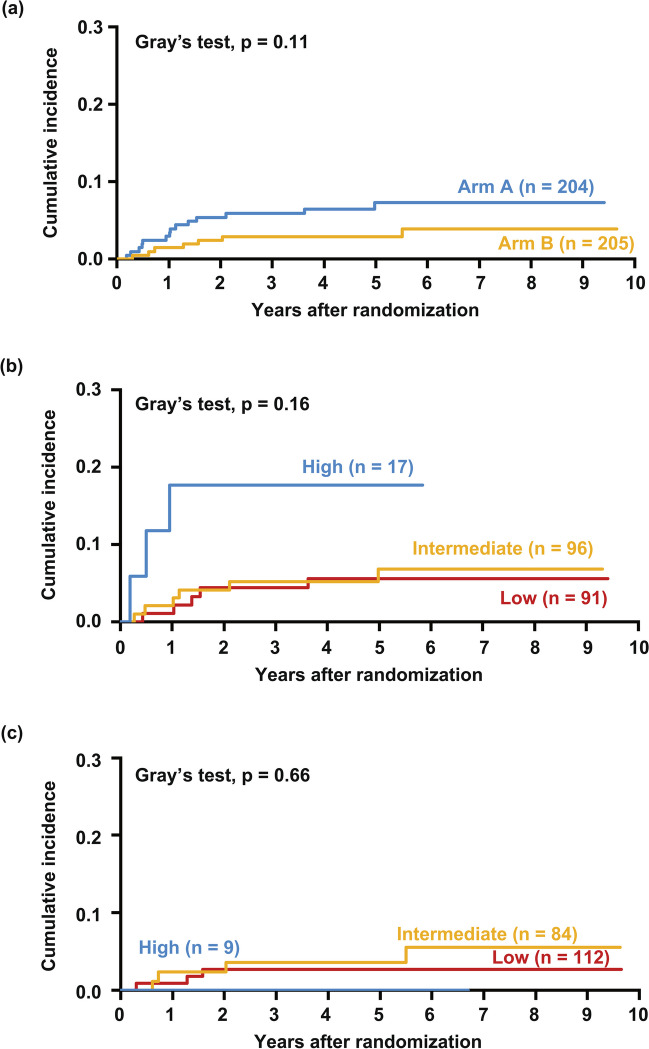
Cumulative incidence of sCNSi according to treatment arm. The cumulative incidence of sCNSi is shown according to treatment arm (**a**), according to CNS-IPI in arm A (**b**), and according to CNS-IPI in arm B (**c**)

Within a median follow-up duration of 4.9 years, 21 (5%) patients developed sCNSi. The 5-year cumulative incidence of sCNSi was 5.1% (95% confidence interval [CI] 3.2–7.6), and that in patients at low, intermediate, and high risk by CNS-IPI was 4.0% (95% CI 1.9–7.4), 5.3% (95% CI 2.6–9.4), and 11.5% (95% CI 2.8–27.1), respectively (Fig. 1 a and b). Of the 21 patients, 17 (81%) developed isolated CNS involvement and the remaining 4 (19%) patients had systemic lesions other than CNS involvement simultaneously. In terms of treatment arm, the 5-year cumulative incidence was 7.3% (95% CI 4.1–11.6) in arm A and 2.9% (95% CI 1.2–5.9) in arm B (Fig. 2 a). In patients in arm A, the 5-year cumulative incidence by CNS-IPI was 5.6% (95% CI 2.1–11.8), 6.8% (95% CI 2.7–13.6), and 17.6% (95% CI 4.1–39.1) in patients at low, intermediate, and high risk, respectively (Fig. 2 b). In patients in arm B, these values were 2.7% (95% CI 0.7–7.0) patients at low risk and 3.6% (95% CI 0.9–9.2) in those at intermediate risk (Fig. 2 c). None of the high-risk patients in arm B had sCNSi. Table 2 lists the characteristics of the 21 patients with sCNSi. The CNS-IPI of these patients was low in 8, intermediate in 10, and high in 3; and 15/21 (71%) patients had extranodal involvement at the time of registration. Extranodal lesion locations found in two or more patients were the stomach (n = 4), paranasal cavity (n = 3), and bone marrow (n = 2). Of the 21 patients, the initial treatment response was progressive disease (PD) in 6 patients, and all but one patient had extranodal involvement. Five patients developed sCNSi at more than 2 years after registration, and three of these five patients did not have any extranodal lesion at the time of registration. Five of eight patients who developed sCNSi despite being low risk by CNS-IPI had extranodal involvement (stomach [n = 1], paranasal cavity [n = 1], nasal cavity [n = 1], thyroid Fn = 1], and breast [n = 1]). The median time to the development of sCNSi was 1 year (range 0.2–5.5 years). That classified by CNS-IPI was 1.3 years (range 0.3–3.6) in low, 1.1 years (range 0.3–5.5) in intermediate, and 0.5 years (range 0.2–0.9) in high-risk patients.

**Table 2:** Characteristics of 21 patients with secondary CNS involvement

	Extnodal sites at registration	IPI	CNS-IPI	Initial response	Pattern of relapse	PFS	OS	OS
						(y)	(y)	events
Arm A (n = 14)	Stomach, bone, bone marrow	H	H	PD	isolated	0.2	3.3	Alive
	-	LI	I	PD	isolated	0.3	0.7	DOD
	Stomach	L	L	PD	with systemic disease	0.4	1.2	Alive
	Lung	HI	I	PD	isolated	0.5	0.8	DOD
	Stomach, skin/subcutaneous	H	H	PD	isolated	0.5	1.2	DOD
	Paranasal cavity, penis	H	H	CR	isolated	0.9	6.3	Alive
	Nasal cavity	L	L	CR	isolated	1.0	4.3	DOD
	Stomach	LI	I	CR	isolated	1.0	1.8	DOD
	Colon	LI	I	CR	with systemic disease	1.1	1.2	DOC
	-	L	L	CR	isolated	1.4	3.8	Alive
	Paranasal cavity	L	L	CR	isolated	1.5	4.2	DOD
	Iliopsoas	HI	I	CR	isolated	2.1	3.1	Alive
	-	L	L	CR	isolated	3.6	4.8	Alive
	-	LI	I	CR	isolated	5.0	6.2	DOC
Arm B (n = 7)	Thyroid gland	L	L	PD	isolated	0.3	0.4	DOD
	Orbital cavity, paranasal cavity	LI	I	CR	with systemic disease	0.6	1.3	DOD
	Skin/subcutaneous	HI	I	PR	isolated	0.7	0.9	DOD
	Breast	L	L	CR	with systemic disease	1.3	3.2	DOD
	-	L	L	CR	isolated	1.6	5.0	Alive
	Bone marrow	HI	I	PR	isolated	2.0	2.6	DOD
	-	LI	I	CR	isolated	5.5	6.0	DOD

Abbreviations: IPI, international prognostic index; CNS, central nervous system; PFS, progression-free survival; OS, overall survival; H, high; HI, high-intermediate; LI, low-intermediate; L, low; I, intermediate; PD, progressive disease; CR, complete response; PR, partial response; DOD, died of disease; DOC, died of other cause

### Prognostic factor analysis

**Fig. 3:**
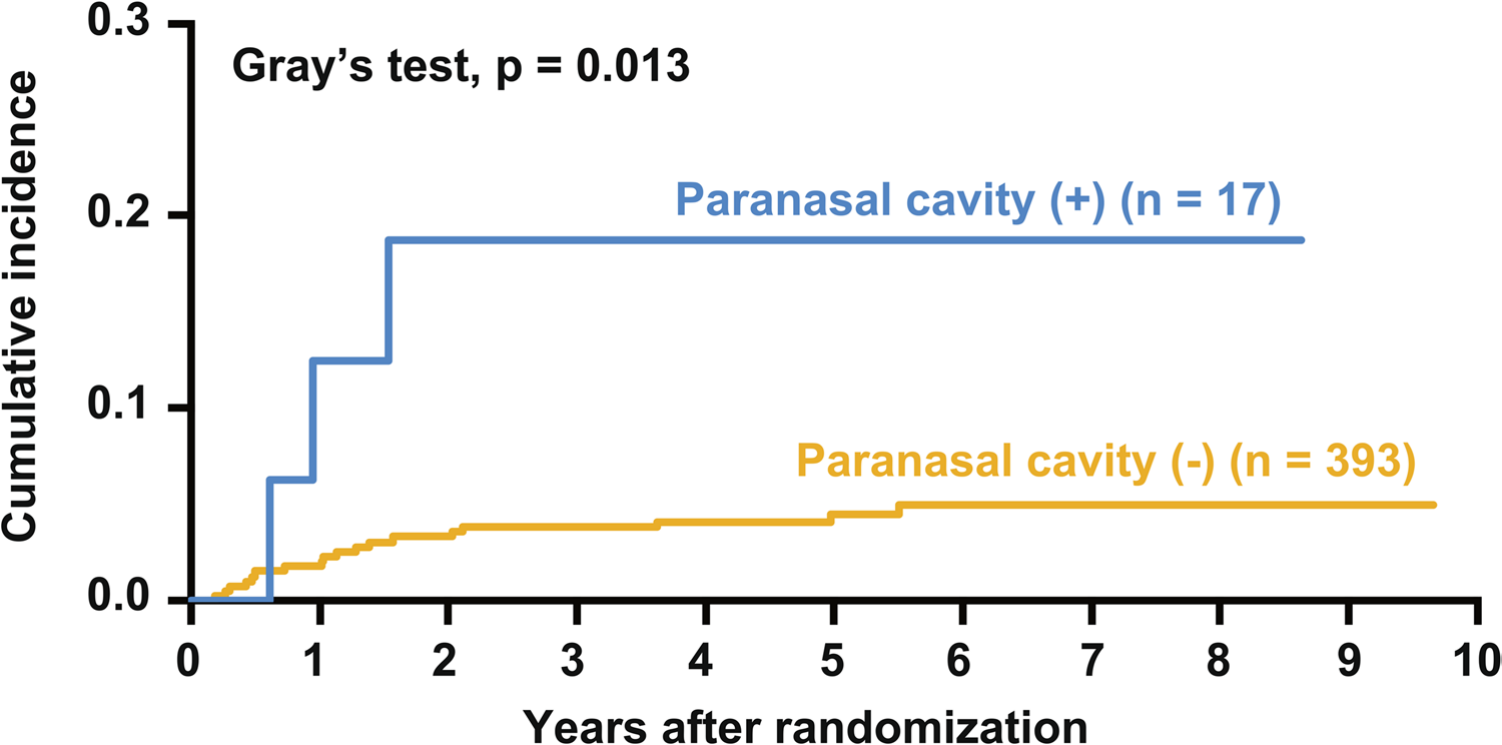
Cumulative incidence of sCNSi according to paranasal cavity involvement

We next analyzed prognostic factors (Table S1). In univariable analysis, paranasal cavity lesion (sHR 4.34, 95% CI 1.28–14.73, p = 0.02), orbital cavity lesion (sHR 14.16, 95% CI 1.61–124.34, p = 0.02), and age > 60 years (sHR 2.79, 95% CI 1.02–7.65 p = 0.046) were high-risk factors for sCNSi. The 2-year cumulative incidence in patients with and without paranasal cavity involvement was 18.8% (95% CI 4.3–41.1) and 3.3% (91%CI 1.9–5.4), respectively (Fig. 3). In terms of cell of origin by Hans classifier, the risk of non-germinal center B-cell (GCB) type was relatively high (sHR 2.89, 95% CI 0.84–9.97, p = 0.094). Two-year cumulative incidence was 1.6% (95% CI 0.3–5.3) in patients with GCB type and 4.8% (95% CI 2.4–8.3) in those with non-GCB type. Kidney, adrenal gland, and testis are well known to be high-risk organs for sCNSi but were not evaluated in the present study because none of these lesions were observed at the time of registration in any patient with sCNSi.

### Prognosis after the development of sCNSi

**Fig. 4:**
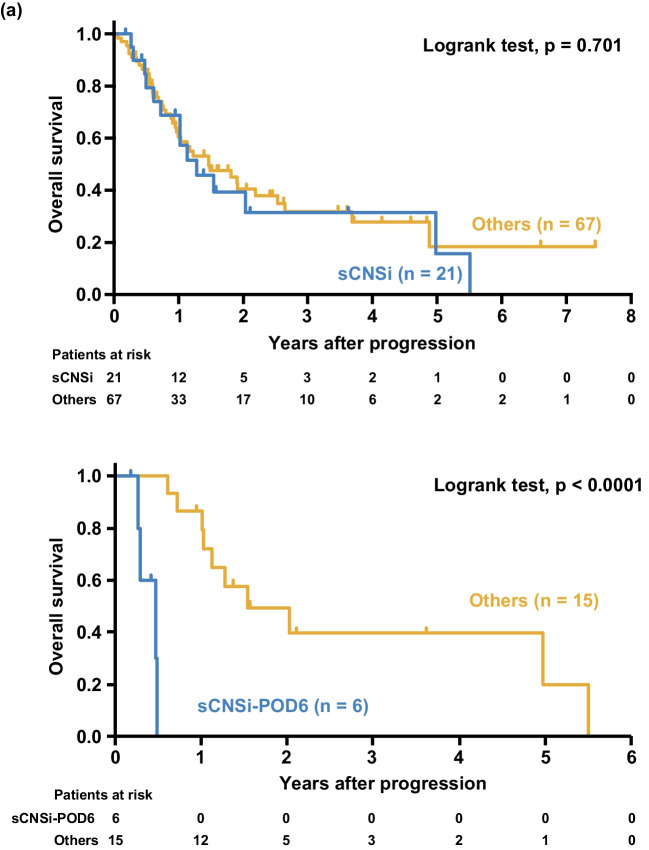
Overall survival after progression according to development of sCNSi. Overall survival is shown after progression in the sCNSi group and in the non-CNS progression or relapse group (**a**), and according to time from registration in the sCNSi group (**b**)

As of the point of data cutoff, 7 of the 21 patients who developed sCNSi survived. Within the median follow-up duration of 3.8 years after the development of sCNSi in the surviving patients, median survival time was 1.3 years, and 2-year OS was 39.3% (95% CI 17.4–60.7). In the 67 patients with non-CNS progression/relapse, median survival time from progression/relapse was 1.5 years, 2-year OS was 40.4% (95% CI 27.3–53.2), and there was no significant difference between the sCNSi group and the non-CNS progression or relapse group (p = 0.70) (Fig. 4 a). Furthermore, median survival time was 5.7 months (95% CI 3.2–5.9) in patients who developed sCNSi within 6 months after registration (sCNSi-POD6) and was 18.5 months (95% CI 12.2–66.1) thereafter (p < 0.0001), which suggests that patients with early development of sCNSi were resistant to subsequent therapies (Fig. 4 b).

## Discussion

The present analysis examined sCNSi in patients treated with rituximab combined with CHOP as a supplementary analysis of JCOG0601, in which most patients were of lower IPI risk. The overall cumulative incidence of sCNSi of 5.1% was comparable with those of previous reports [1–5]. Of 21 patients who developed sCNSi, 8 and 10 were categorized as low and intermediate risk by CNS-IPI, respectively, and lesions in the paranasal cavity and orbital cavity were identified as having significantly high risk. Age > 60 years was also identified as a high-risk factor. In general, OS after sCNSi has been reported to be poor [4, 9], but the 2-year OS of 39.3% after the development of sCNSi was similar to that of non-CNS progression or relapse in JCOG0601.

The JCOG0601 compared standard R-CHOP-21 (arm A) with RW-CHOP-21 (arm B). The study demonstrated that PFS as the primary endpoint did not differ between the two arms, and could not confirm the superiority of dose-dense administration of rituximab at the start of treatment [18]. It is noteworthy that the number of patients with sCNSi was lower in arm B than in arm A, which requires careful interpretation. Compared to patients in arm A, many more patients in arm B were lower IPI risk (88% vs 76%), fewer were high IPI risk (4% vs 7%), and fewer were high CNS-IPI risk (4% vs 8%). Although the lack of patients with sCNSi in the high risk category of CNS-IPI in arm B might have led to this difference, the dose-dense administration of rituximab might have led to the lower number of patients with sCNSi in arm B.

In general, sCNSi in DLBCL had developed by 2 years after diagnosis [2, 5, 20]. In the present analysis, the time from registration to sCNSi by CNS-IPI ranged from 0.5 to 1.3 years among the risk categories, and the time to sCNSi was shortest in patients with high risk by CNS-IPI. These durations are comparable with those in previous studies [2, 20]. Intriguingly, three of the five patients who developed sCNSi at more than 2 years after registration did not have extranodal involvement at the time of registration. A possible explanation for this finding might be that a longer duration would be required for tumor cells in lymph nodes to develop a CNS lesion. In contrast, patients with early development of sCNSi, and particularly those with sCNSi-POD6, might have had subclinical CNS involvement at the time of registration. Clinical outcomes of these patients were extremely poor, similar to those reported in a recent study [21]. Given that patients with early-onset sCNSi developed the disease during the initial series of treatment and were quite resistant to subsequent therapies, biological differences in the nature of lymphoma cells between patients with early development and those with late onset should be investigated in the future.

The present analysis identified lesions in the paranasal cavity and orbital cavity as high risk for sCNSi. According to previous studies, the paranasal cavity is well known to be a high-risk organ [22–24], and the fact that sCNSi can occur in lower-risk patients by CNS-IPI might be associated with the proximity of the paranasal cavity and CNS. The orbital cavity is also well known as a high-risk organ [25]; however, only 1 of 2 patients with such a lesion developed sCNSi, which is too few to enable interpretation. We were also unable to confirm the significance of lesions in the kidney, adrenal gland, and testis, which are known to be high-risk organs [14, 20], because no patient with sCNSi had a lesion in these organs at the time of registration. This might be related to the low frequency of lesions occurring in these organs and because the study initially targeted patients with lower IPI risk.

In the present analysis, outcomes of patients with sCNSi and non-CNS progression were comparable. In previously reported analyses, outcomes of patients with non-CNS progression were extremely poor for the strategy of conventional salvage therapies plus high-dose therapy with autologous stem cell transplantation, especially in patients who were refractory to the initial series of treatment or who had relapsed disease within 12 months [26, 27]. Outcomes in these patients have shown remarkable improvement, from approximately 20% to 50%, by applying chimeric antigen receptor (CAR) T-cell therapy [28–31]. In contrast, in patients with sCNSi, several prospective studies have indicated that the outcomes of patients who could receive high-dose therapy with autologous stem cell transplantation after high-dose methotrexate-based regimen were comparable to those of patients with non-CNS progression [32–34]. This suggests that active treatment is desirable for younger patients with sCNSi who respond to salvage therapies. In terms of elderly patients, CAR T-cell therapy is becoming widely applied to patients with non-CNS progression. However, the effectiveness of CAR T-cell therapy is limited in patients with sCNSi [35]. Further development of treatment for elderly patients is required.

Finally, we should discuss the limitations of this analysis. The present study is a supplementary analysis based on the JCOG0601, which ensured a uniform population and protocol treatment. However, sCNSi data were available only at the first progression or at relapse, and were not available after the second or later progression or relapse. Therefore, the overall cumulative incidence might have been underestimated. In addition, information regarding treatment after sCNSi was insufficient, despite the clear importance of treatment after sCNSi. Nonetheless, the present study could provide basic information regarding sCNSi.

## Conclusions

In conclusion, although the risk of sCNSi in patients at lower risk by CNS-IPI who were treated with rituximab combined with CHOP was low, it is important to pay attention to the development of sCNSi in patients with paranasal cavity lesions, even in those at lower risk by CNS-IPI.

### Supplementary Information

Below is the link to the electronic supplementary material.

Supplementary file1 (XLSX 12 KB)

## Data Availability

Individual participant data that underlie the results reported in this article will not be shared because the follow-up of the patients is continued until Dec. 2022. After the publication using data as of Dec. 2022, individual participant data that underlie the results after deidentification will be shared if investigators whose proposed use of the data has been approved by the investigators from Lymphoma Study Group of JCOG identified for this purpose. Proposals should be directed to 8jmmd004@is.icc.u-tokai.ac.jp. The data will be available for achieving aims in the approved proposal.
